# Sodium alginate and collagen-loaded troxerutin improve mineralization and vascularization around implants

**DOI:** 10.3389/fbioe.2026.1935336

**Published:** 2026-10-19

**Authors:** Zhe-Zhen Lin, Yan-Jing Ou, Xi Ding, Jiang Chen

**Affiliations:** 1 School and Hospital of Stomatology, Fujian Medical University, Fuzhou, China; 2 Department of Stomatology, The First Affiliated Hospital of Wenzhou Medical University, Wenzhou, China

**Keywords:** angiogenic, collagen, osteogenic, titanium, troxerutin

## Abstract

Mineralization and vascularization are essential processes for implant osseointegration around titanium, and insufficient surface bioactivity affects its clinical performance; thus, surface modification of implants is necessary. This study aimed to establish a sodium alginate/collagen/troxerutin coating on the surface of titanium to promote angiogenic and osteogenic ability. The results indicate that the material has satisfactory biocompatibility in terms of cell viability. Mouse embryonic osteoblast cells (MC3T3-E1) cells on the alginate/collagen/troxerutin coating significantly influenced mineralized nodule formation and Alkaline phosphatase (ALP) activity. The alginate/collagen/troxerutin coating promoted tube formation and the migration of human umbilical vein endothelial cells (HUVECs). The alginate/collagen/troxerutin coating also resulted in increased osteogenic and angiogenic gene expression. In addition, more bone formation was observed in the Ti-alginate/collagen/troxerutin group than in the other groups *in vivo*. The combination of troxerutin with sodium alginate and collagen resulted in osteogenic and angiogenic effects. It offers a potential option for surface modification of implants and has good application prospects in the future.

## 1 Introduction

Titanium is recognized as a component with good biocompatibility (Ogawa et al., 2025). Implant osseointegration is crucial for the implant survival rate. However, titanium lacks osteoconductivity and osteoinductivity, which are essential for implant osseointegration (Chang et al., 2024). Incomplete osseointegration can cause implant failure, especially in patients with systemic diseases, such as osteoporosis and diabetes (Sachelarie et al., 2025). After implant insertion, remodeling around the implants occurs, and this process involves a series of events, including osteogenesis, angiogenesis, and stem cell homing. Angiogenesis and osteogenesis are important processes involved in remodeling around implants. Blood vessels promote hematoma organization, callus formation and bone tissue reconstruction by providing oxygen, nutrition and cell support (Liu et al., 2025).

It is difficult to meet the different demands of bone formation by relying solely on functional materials. Surface modification is considered a proper way to implement biological strategies. Surface modification can involve chemical, topographic, or mechanical properties (Raju and Biswas, 2025). When the topography is changed, the surface toughness is influenced, which can affect platelet adhesion and activation (Kouveliotis et al., 2024; Jesus et al., 2024). Chemical modification can directly participate in cell adhesion, migration, proliferation, and differentiation (Kim et al., 2016). Chemical surface modification is performed mainly through wet chemical modification, including silanization, silylation, and acetylation. The main problem with chemical modification is the toxicity of organic substances.

Polyphenols can be used for chemical modifications. Natural polyphenols can protect against cardiovascular disease (Li et al., 2024). Polyphenols have various effects, including osteogenic ability, antibacterial activity, angiogenesis, and the ability to alleviate cellular oxidative stress (Wu et al., 2022; Jin et al., 2023; Zheng et al., 2025). Researchers have established polyphenol coatings on titanium that can be released slowly; these coatings have potential for clinical application (Morra et al., 2024; Su et al., 2025; Sun et al., 2025). However, few studies have combined titanium and troxerutin. Troxerutin is a kind of polyphenol, which is used in treating cardiovascular and cerebrovascular diseases in clinic. Troxerutin can also enhance the osteogenic ability of materials (Wei et al., 2025). Yang et al. (2021) found that troxerutin could promote osteogenic ability *in vitro* and *in vivo*. And the troxerutin needs to be delivered by a carrier. Sodium alginate and collagen is a common carrier in biomedical materials (Fu et al., 2026; Yue et al., 2026; Hua et al., 2026). Sodium alginate is a kind of natural polysaccharide, which has hydrophilicity and excellent biocompatibility, and could be used for encapsulating compounds (Chen et al., 2026; Fang et al., 2026).

In our study, to achieve enhanced osseointegration, our approach involved surface modification of titanium using troxerutin, sodium alginate and collagen. We aimed to identify the osteogenic ability and vascularization ability around this material using mouse embryonic osteoblast cells (MC3T3-E1) and human umbilical vein endothelial cells (HUVECs). We believe that the method presented in our study can contribute to the osseointegration process of titanium, which is a challenge in implantology.

## 2 Materials and methods

### 2.1 Material preparation

Titanium discs were purchased from China Fuxin. Ltd., Baoji. Titanium (10 mm diameter, 1 mm thickness) was cleaned with acetone, ethanol and distilled water. Then, the titanium was treated with a sodium hydroxide solution for 24 h at 80 °C (0.2 g/mL; Maclin, Biochemical Technology, Shanghai, China). We used layer-by-layer method in this procedure. Titanium was immersed in a mixture of collagen (0.02 g/mL, Maclin, Biochemical Technology, Shanghai, China) and sodium alginate (2 mg/mL, Aladdin Biochemical Technology, Shanghai, China) for 30 min. In addition, the titanium was immersed in a troxerutin solution (0.2 mg/mL, Maclin, Biochemical Technology, Shanghai, China) for 24 h at 37 °C. The titanium was subsequently dried and disinfected (ultraviolet disinfection for 12 h) for subsequent cell and animal experiments (Figure 1).

**FIGURE 1:**
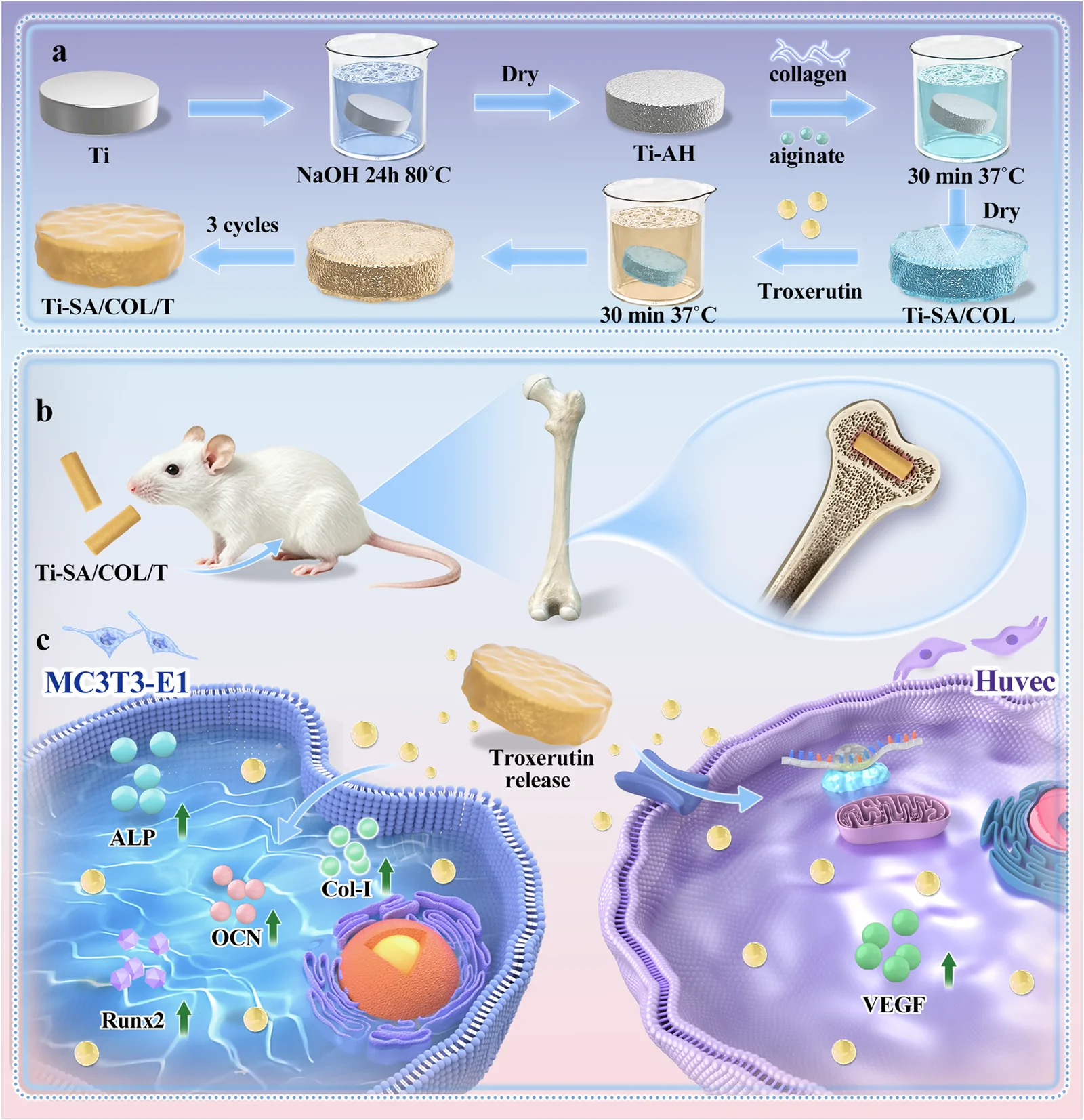
**(A)** Preparation scheme of the Ti-SA/COL/T coating; **(B)** Representative image of experiment *in vivo*; **(C)** Experiment *in vitro*.

### 2.2 Surface structure

Surface morphologies were recorded using scanning electron microscopy (SEM; ZEISS Sigma 300, Germany), and interfacial wettability was measured according to water contact angle measurements (Ding Sheng, Cheng De, China). To measure the chemical composition of Ti, X-ray diffraction (XRD) patterns were measured using an X-ray diffractometer (Bruker D8 Advance, Germany). Atomic force microscopy (AFM) revealed the three-dimensional morphology of the materials (Bruker Dimension Icon, Germany). The scanning range was 5 μm × 5 μm.

### 2.3 Release of troxerutin *in vitro*

*In vitro* release experiments were carried out by simulating the environment of the body. 50 mL Fresh phosphate-buffered saline (PBS) was used in the drug release experiment at 37 °C via the Franz diffusion method at 100 r/min. Every 2 days of total 14 days, 100 μL solution was used for the following test. A UV‒Vis spectrophotometer at a wavelength of 290 nm was used to quantify drug release. Every experiment was performed three times.

### 2.4 *In vitro* biocompatibility experiments

MC3T3-E1 cells were cultured in α-minimum essential medium (α-MEM) under standard conditions (37 °C, 5% CO_2_, humidified incubator) supplemented with 1% penicillin/streptomycin and 10% FBS (fetal bovine serum). HUVECs were cultured in high-sugar Dulbecco’s Modified Eagle Medium (DMEM) under standard conditions (37 °C, 5% CO_2_, humidified incubator) supplemented with 1% penicillin/streptomycin and 10% FBS (fetal bovine serum). The medium was changed every 2 days. We used a cell counting kit (CCK-8) standard protocol to evaluate cell proliferation after 4 days and 7 days. The intensity of the color reaction was measured according to the absorbance at 450 nm with a spectrophotometric microplate reader (MEIGU, United States). Independent one-way ANOVA was performed between different groups.

### 2.5 Osteogenic ability

#### 2.5.1 Alkaline phosphatase (ALP) activity

Alkaline phosphatase activity was measured by an ALP Assay Kit after 7 days (JianCheng, Nanjing, China). All the samples were immersed in 1% Triton X-100 (60 min, 4 °C), the product of each well was collected, and the OD value was recorded at 520 nm. A BCA protein assay kit was also used, and the OD value was recorded at 562 nm (Biyotime, Shanghai, China). The results were normalized to the total protein concentration.

#### 2.5.2 Alizarin red staining

After 21 days, calcification was detected by alizarin red staining (ARS). The MC3T3-E1 cells were treated with a 10% formaldehyde solution for 60 min at 4 °C. The samples were stained with ARS solution (Oricell, Guangzhou, China). Afterward, further quantitative analysis at 562 nm was performed using 10% cetylpyridinium chloride (Maclin, Biochemical Technology, Shanghai, China).

### 2.6 Angiogenic ability

#### 2.6.1 *In vitro* tube formation

Tube formation assays were carried out to assess the angiogenic ability of the materials. An indirect noncontact model was used in this study. The Matrigel was thawed overnight, placed into the bottom of a prechilled 24-well Transwell plate with a 0.4 μm pore filter, and then stored at 37 °C for 1 h (Biyotime, Shanghai, China). HUVECs (1 × 105 cells per well) were inoculated on the surface of Matrigel for 6 h at 37 °C, and the materials were placed at the top of a 24-well Transwell plate (Corning, New York, United States). An inverted light microscope was used to detect tube formation. Three random fields at the ×10 magnification were acquired for each group. Quantitative analysis was performed according to the node number.

#### 2.6.2 Cell migration assay using a transwell plate

To form a chemotactic gradient, high serum culture medium (1,000 µL) and materials were added to the bottom chamber. Cells (5 × 104 cells per well) with low serum culture medium were added to an insert with an 8 μm porous membrane and incubated for 24 h at room temperature. After 24 h, the insert was fixed with 4% paraformaldehyde for 30 min and immersed in 0.1% crystal violet solution for 1 h (BKMAM, Changde, China). Nonmigrated cells were removed from the apical side of the insert membrane. The migrated cells were fixed on the basal side of the insert membrane. An inverted light microscope was used to detect migrated cells. Three random fields at the ×10 magnification were acquired for each group.

### 2.7 Gene expression

Total RNA was extracted from MC3T3-E1 cells (cultured for 7 days) and HUVECs (cultured for 4 days) using a FastPure Cell Total RNA Isolation Mini Kit (Vazyme, Nanjing, China). We used a HiScript III RT SuperMix for qPCR (Vazyme, Nanjing, China) to obtain cDNA for reverse transcription. Real-time polymerase chain reaction (RT‒PCR) was conducted using Vazyme qPCR SYBR® Green Master Mix to measure the gene expression levels of COL1A1, ALPL, RUNX2, BGLAP, VEGF, and an endogenous control gene (glyceraldehyde-3-phosphate dehydrogenase, GAPDH). A NanoDrop One (Thermo Fisher, United States) was used to measure RNA integrity and concentration. mRNA expression was determined using the 2^−ΔΔCT^ method. The sequences of the PCR primers used are listed in Supplementary Tables S1 and S2. Three technical replicates were performed in this section.

### 2.8 Construction of a rat implant model

The animal experiments were approved by the Ethics Committee of The First Affiliated Hospital of Wenzhou Medical University, and all the experiments met the requirements of the Ethics Committee of Wenzhou Medical University. To evaluate osteogenic ability *in vivo*, samples were inserted into the femurs of the rats. These 18 rats were randomly divided into three groups: (1) Ti (titanium), (2) Ti-SA/COL (titanium- alginate/collagen), and (3) Ti-SA/COL-T (titanium- alginate/collagen/troxerutin). The rats were fed clean water and pellet food. For every implant surgery, 5% isoflurane gas with O_2_ was used to induce anesthesia, and this gas was subsequently maintained at 2.5%. A 10 mm-long vertical incision was made, followed by blunt dissection of the muscles and subcutaneous tissue to expose the structure. A 1.0 mm-diameter and 5 mm-long circular hole was drilled into a dental bur, and the surgical area was thoroughly rinsed with normal saline. The wound was sutured with absorbable sutures. After surgery, we checked the wound daily to prevent bleeding and infection. At the end of 4 weeks, the rats were euthanized with sodium thiopental (90 mg/kg intramuscularly), and the femurs were removed for micro-CT scanning to assess bone formation around the implants (Skyscan 1276, Bruker, Kontich, Belgium). CTAn software was used for analysis. The 0.5 mm area around the implant was considered as the region of interest (ROI). The voltage is 60 kV and the current is 200 μA. The same thresholding rules were used in the analysis of micro-CT scanning.

### 2.9 Histological examination

Femurs were harvested for histological examination. The samples were fixed in 4% formalin for 3 days and dehydrated with different concentrations of alcohol. Decalcification was performed using 10% EDTA, and the samples were immersed in paraffin. New bone formation was detected by Masson’s trichrome staining and hematoxylin and eosin (H&E) staining. Immunofluorescence staining was performed to investigate osteogenic and angiogenic ability.

### 2.10 Statistical analysis

Significant differences among the three independent groups were determined by one-way ANOVA, and *post hoc* Tukey’s test was used to compare the differences between the groups. Statistical differences are denoted by *P < 0.05 and **P < 0.01. Levene test was performed in the homogeneity of variance, Welch ANOVA test was used when P < 0.05. The data were analyzed using SPSS 21.0 (New York, United States) and GraphPad InStat® v.3.01 (San Diego, CA, United States). Data are expressed as the mean ± standard deviation (SD).

## 3 Results

### 3.1 Surface structure

The SEM profiles of the different types of titanium are shown in Figure 2A, and we observed a porous structure in Ti-SA/COL, which is beneficial for later drug loading. Troxerutin particles were successfully loaded in Ti-SA/COL-T. The hydrophilicity of the titanium improved after surface modification according to the contact angle (Figures 2B,C). In addition, AFM was used to determine the surface morphology, and heterogeneity was observed after surface modification (Figure 3A). The results of the XRD analysis are shown in Figure 3B. Every group showed the highest orientation peak at 2θ = 70.72, in addition to diffraction index values of 53.22 and 40.39. An additional peak is at 2θ = (32.16) was observed in Ti-SA/COL-T. During the first 10 days, troxerutin underwent an initial burst release (Figure 3C). Subsequently, a slower release occurred.

**FIGURE 2:**
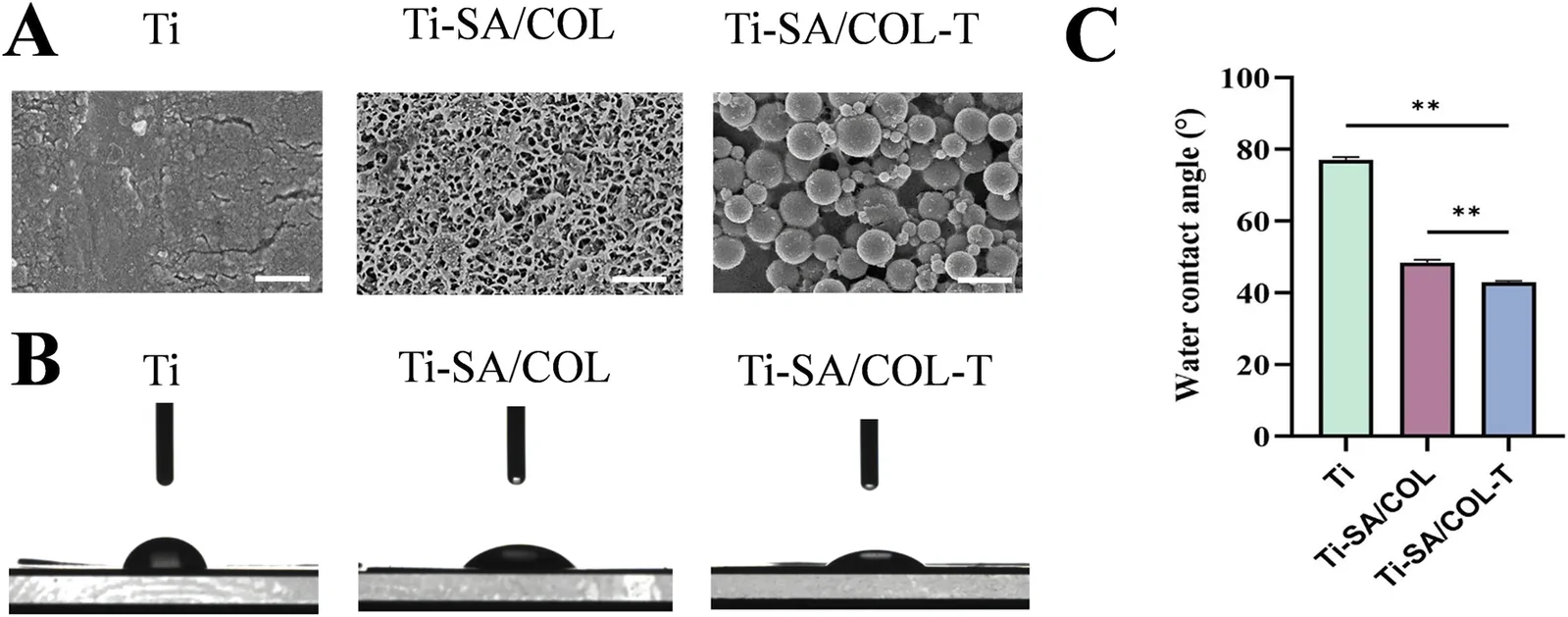
**(A)** SEM images of different types of titanium; **(B, C)** water contact angles of different types of titanium. *p < 0.05, **p < 0.01.

**FIGURE 3:**
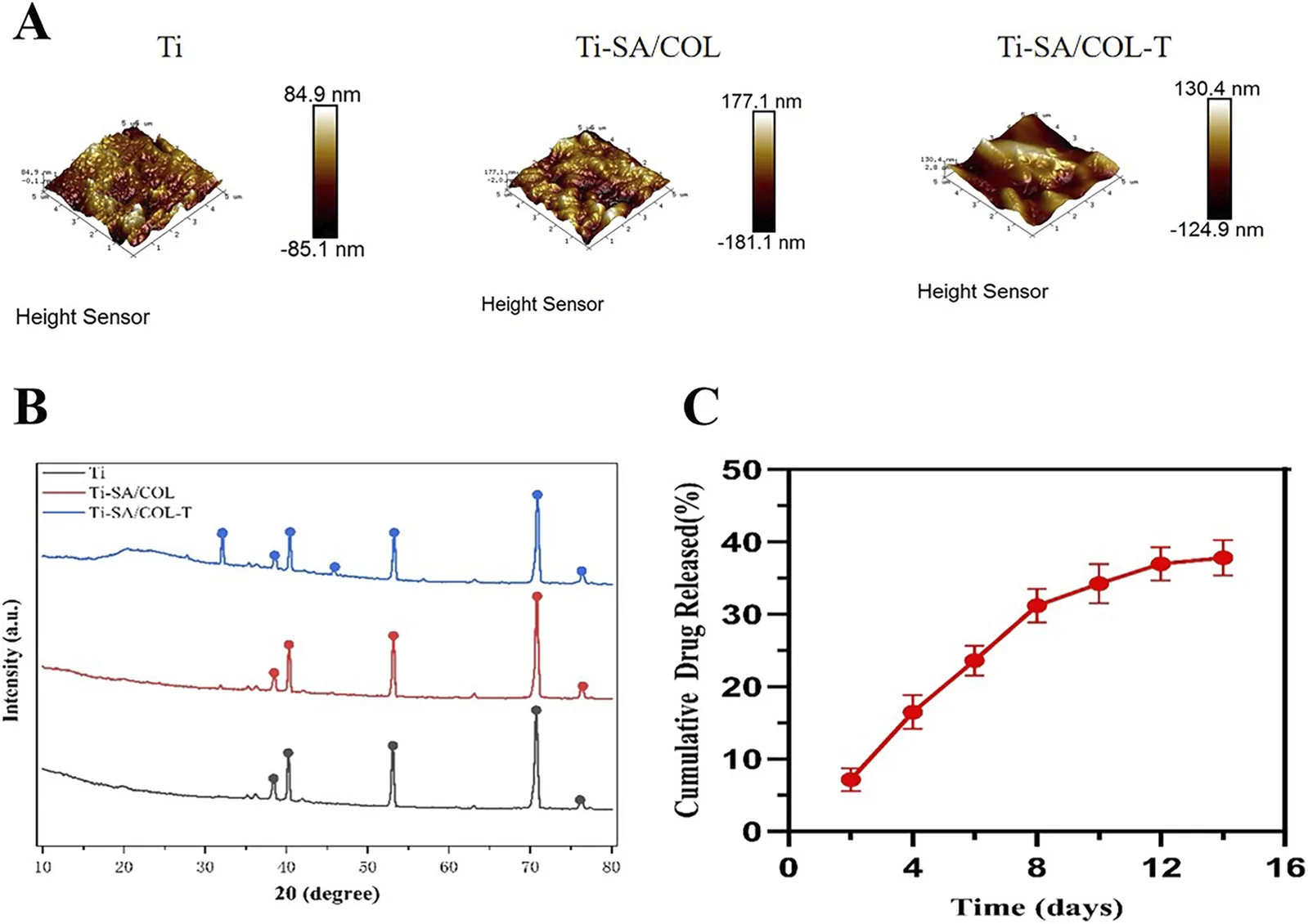
**(A)** AFM images of different types of titanium; **(B)** XRD diffractograms of different types of titanium; **(C)** cumulative release of troxerutin.

### 3.2 Biocompatibility experiments

Titanium biocompatibility was measured according to cell viability and morphology (Figure 4). Compared with the other groups, the Ti-SA/COL-T group exhibited greater cell proliferation on Days 4 and 7. The cell viability of Ti-SA/COL was similar to that of Ti. These results indicated that this coating promoted the viability of MC3T3-E1 cells and HUVECs, which was beneficial for early osteogenic differentiation and angiogenesis.

**FIGURE 4:**
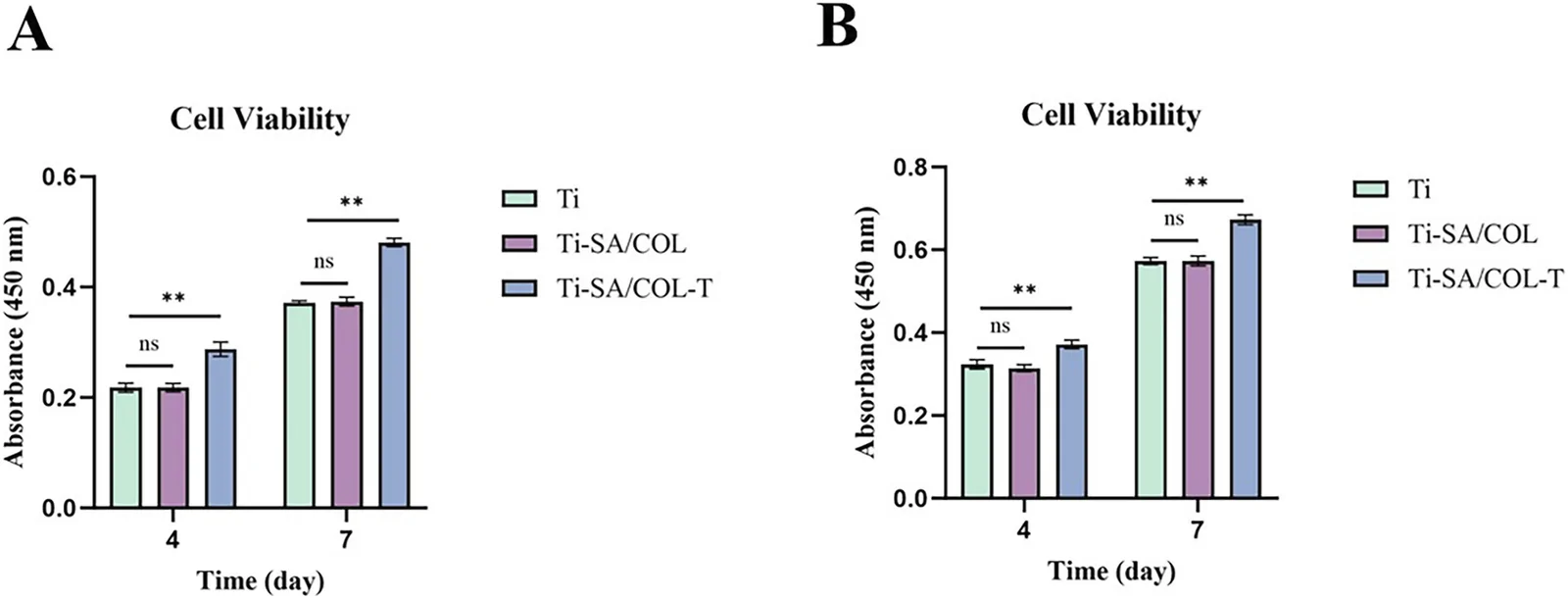
**(A)** Viability of MC3T3-E1 cells was measured by a CCK-8 assay; **(B)** the viability of HUVECs was measured by a CCK-8 assay. *P < 0.05; **P < 0.01.

### 3.3 Osteogenic ability *in vitro*

To confirm the osteogenic ability of this material, we determined ALP activity and conducted alizarin red staining and qRT‒PCR. As shown in Figure 5A, Ti-SA/COL-T obtained the highest level of ALP. Moreover, alizarin red staining revealed that troxerutin enhanced osteogenic differentiation in MC3T3-E1 cells (Figures 5B,C). After the cells were cultured on different coatings for 7 days, the mRNA expression levels of osteogenic differentiation-related genes, including RUNX2, ALPL, BGLAP, and COL1A1, were measured by real-time PCR. Compared with the other groups, the Ti-SA/COL-T group had higher osteogenic mRNA expression levels (Figures 5D–G; P < 0.05).

**FIGURE 5:**
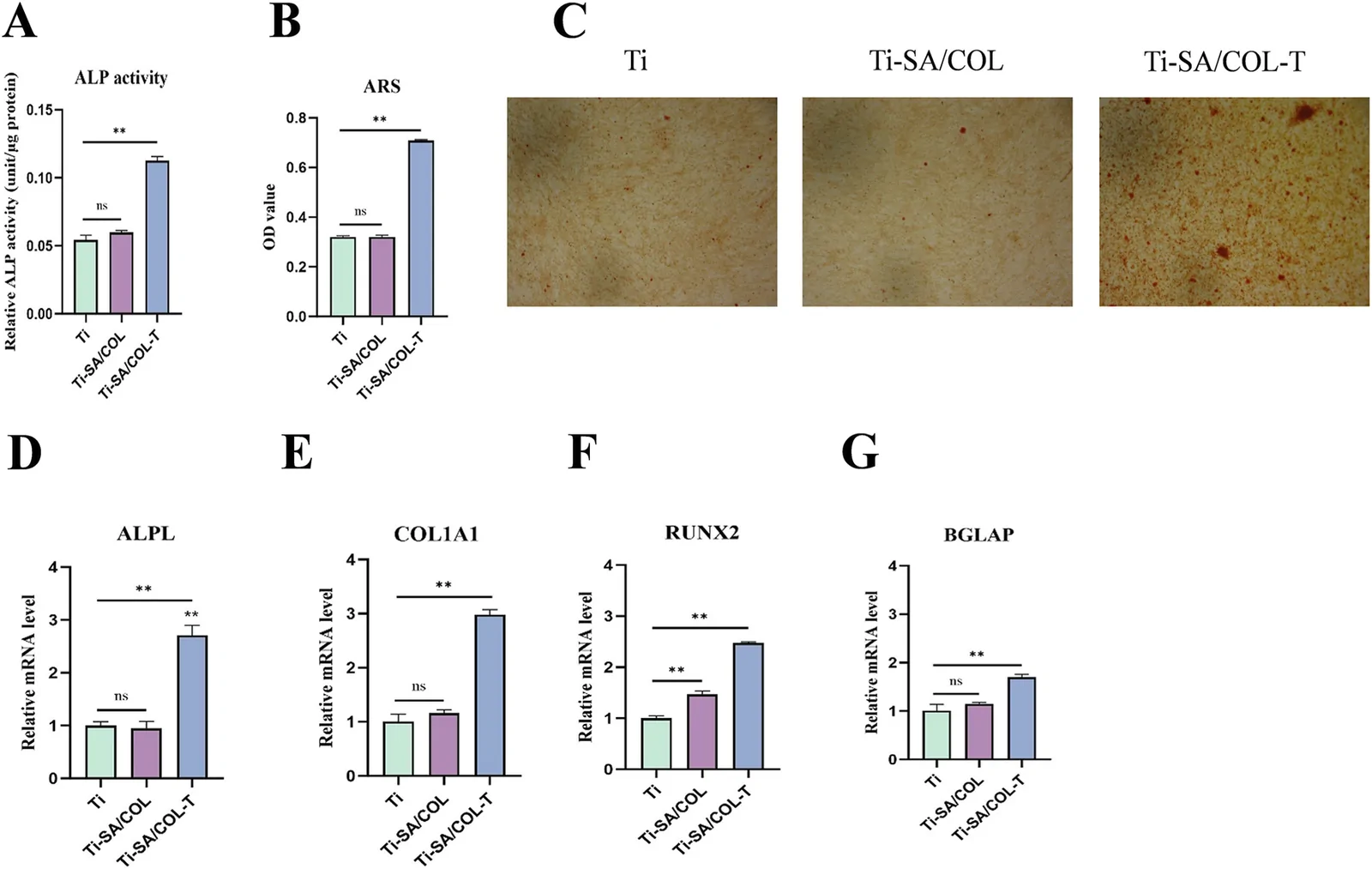
**(A)** Relative ALP activity after 7 days; **(B)** quantitative analysis of alizarin red staining; **(C)** alizarin red staining of MC3T3-E1 cells after osteogenic differentiation for 21 days. **(D)** Relative mRNA level of the ALPL gene; **(E)** relative mRNA level of the COL1A1 gene; **(F)** relative mRNA level of the RUNX2 gene; **(G)** relative mRNA level of the BGLAP gene. *P < 0.05; **P < 0.01.

### 3.4 *In vitro* angiogenesis assay

Tube formation assays and Transwell cell migration assays were performed to evaluate angiogenic ability. Transwell cell migration assays revealed that Ti-SA/COL-T promoted the migration ability of HUVECs, and crystal violet staining was performed after 24 h (Figure 6). These results revealed that Ti-SA/COL-T cells exhibit chemotaxis toward HUVECs. In addition, the performance of Ti-SA/COL-T was better in the tube formation assay (Figure 6). HUVECs were cultured for 6 h, and the formation of tube structures was measured by ImageJ. The tube length in Ti-SA/COL-T was the longest. Moreover, compared with the other groups, the Ti-SA/COL-T group presented higher VEGF gene expression levels (Figure 6).

**FIGURE 6:**
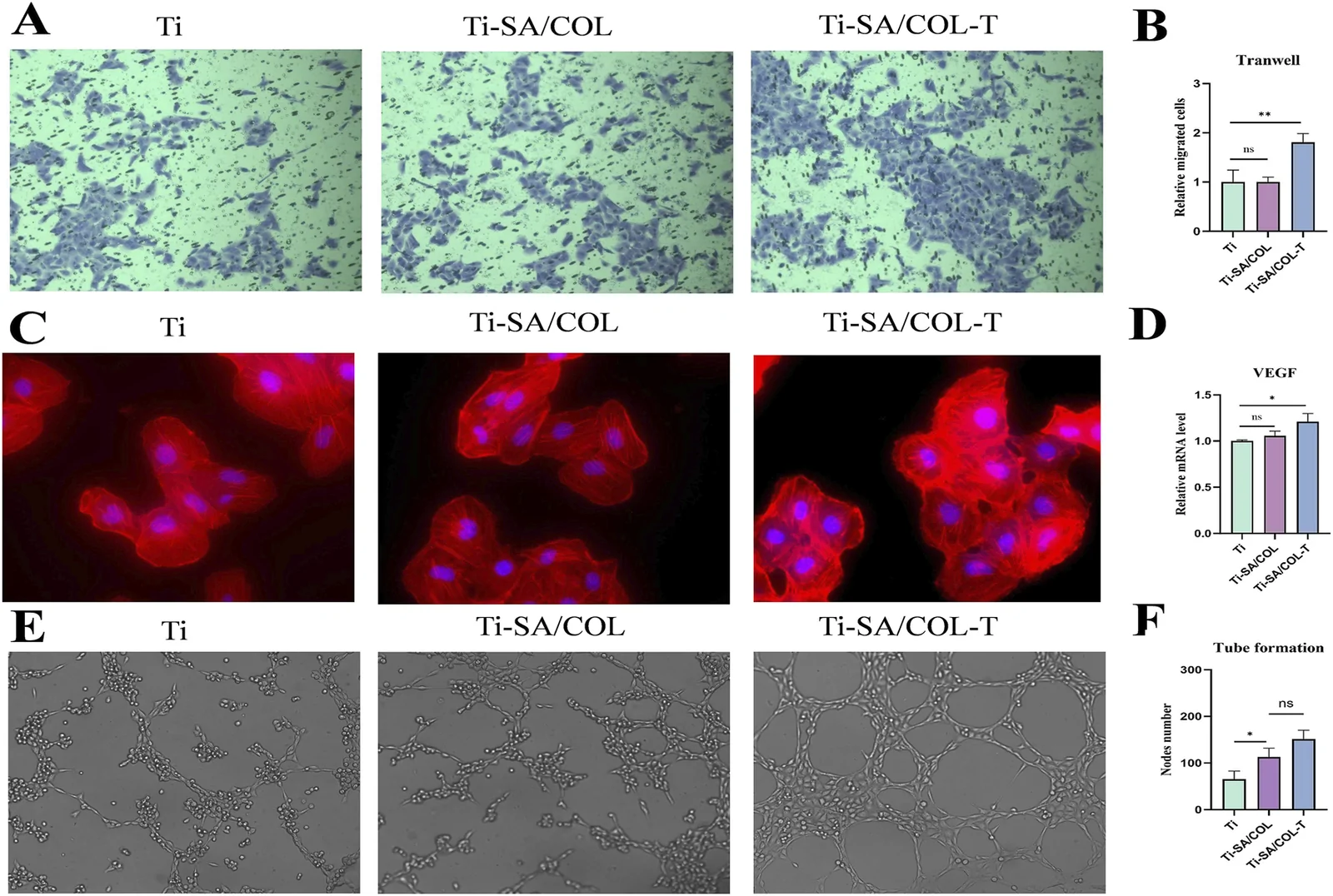
**(A)** Representative image of the Transwell migration assay; **(B)** relative migrated cells in transwell migration assay; **(C)** cytoskeleton of HUVEC cells; **(D)** relative mRNA level of the VEGF gene; **(E)** Representative image of tube formation; **(F)** Quantitative analysis of the tube formation assay. *P < 0.05; **P < 0.01.

### 3.5 *In vivo* experiment

Bone regeneration around the implant was quantified to assess the effect of the SA/COL-T coating. Osteogenesis around the implant increased significantly with troxerutin loading. The bone volume fraction (BV/TV) ratio revealed that Ti-SA/COL-T increased the bone volume around the implants (Figure 7). The quantitative trabecular thickness (Tb.Th) indicated that the Ti-SA/COL-T group presented the highest Tb.Th, outperforming the Ti-SA/COL and Ti groups. H&E and Masson staining revealed greater amounts of newly formed bone tissue around the implants on Ti-SA/COL-T (Figure 8). In addition, more positive signal of Col-1, OCN and OPN was found in immunofluorescence staining (Figure 9).

**FIGURE 7:**
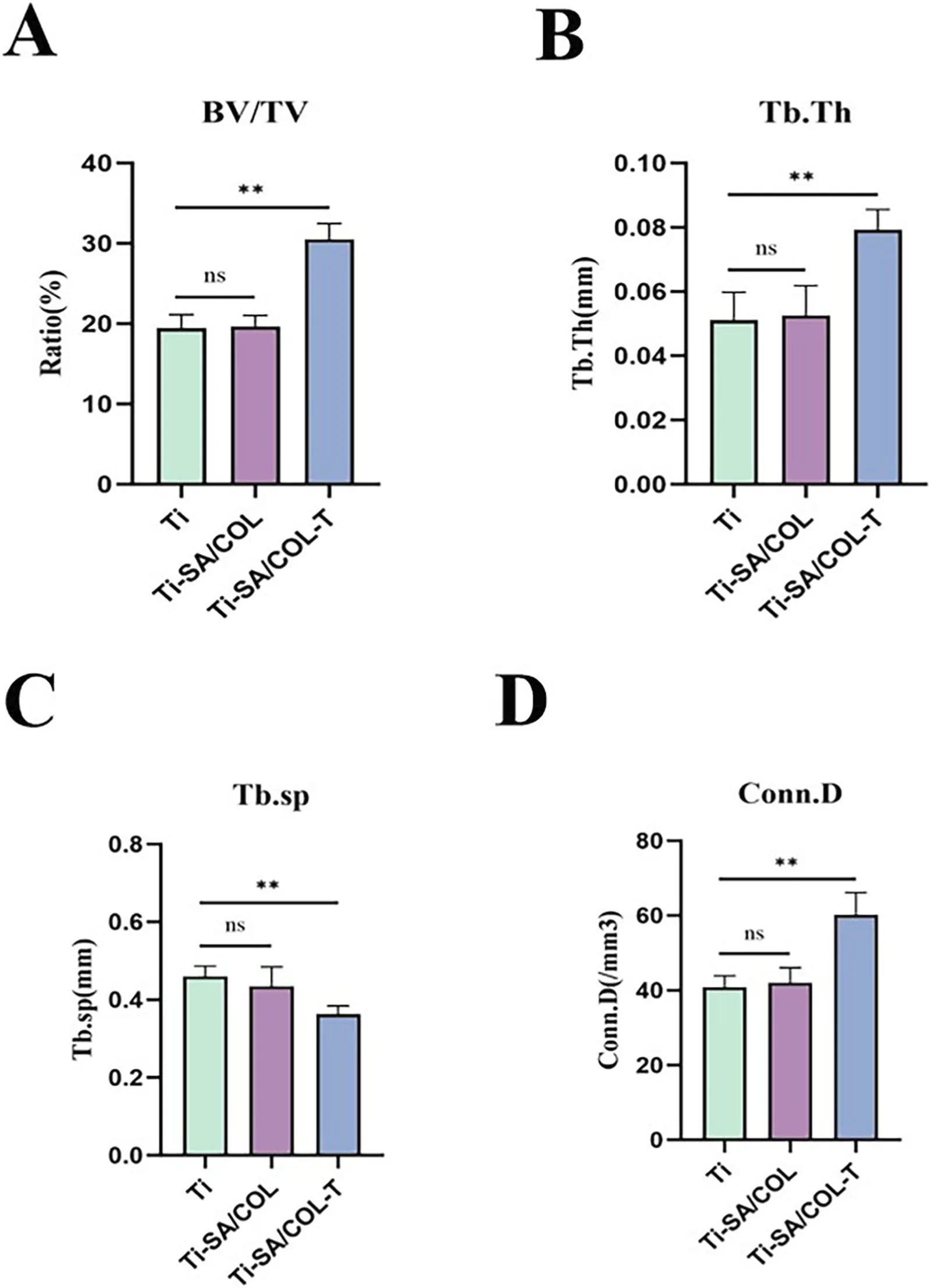
**(A)** BV/TV ratio around the implant in the microct; **(B)** Tb-Th around the implant in the microct; **(C)** Tb. sp around the implant in the microct; **(D)** density of trabecular connections around the implant in the micro-ct. *P < 0.05; **P < 0.01.

**FIGURE 8:**
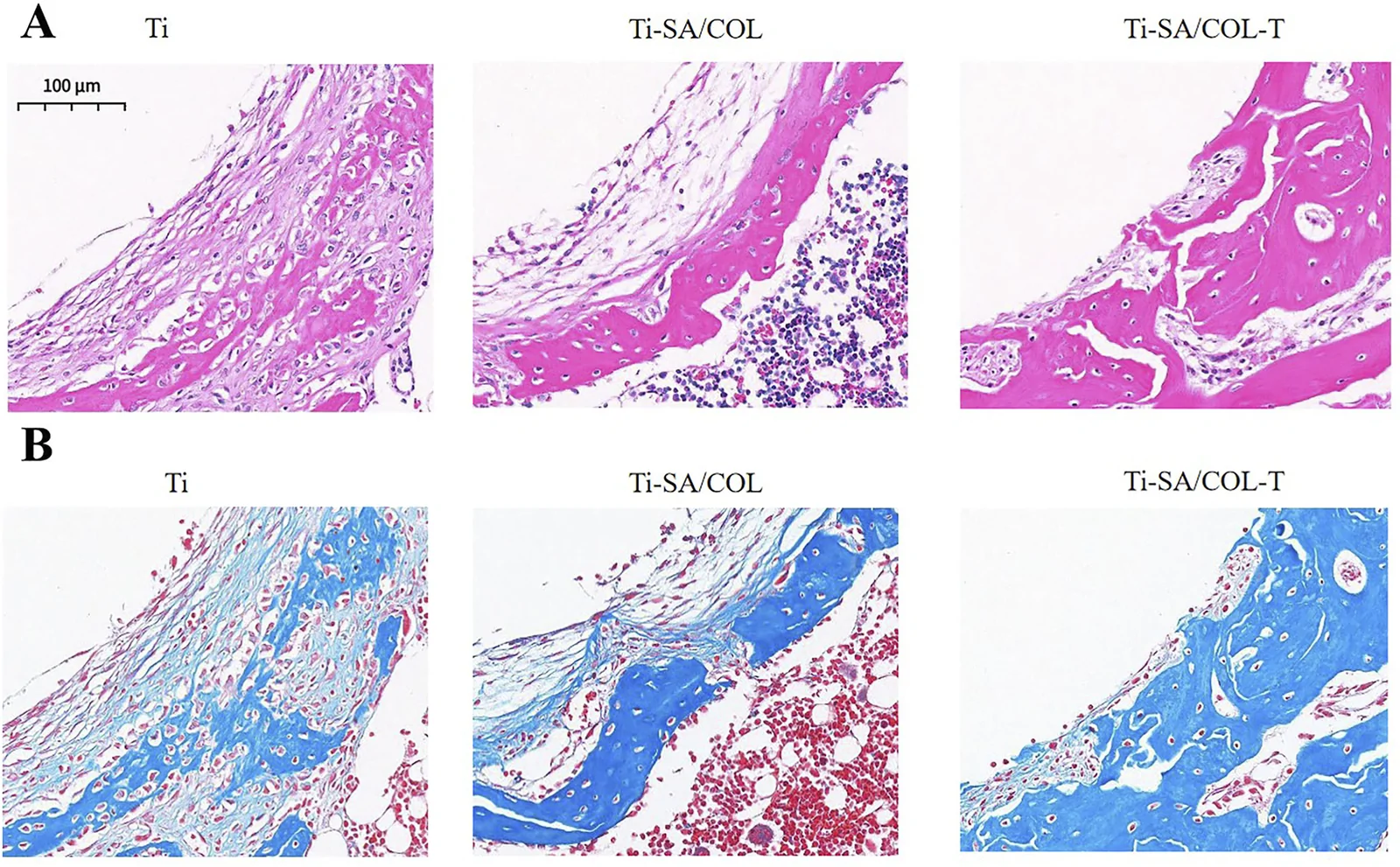
**(A)** Representative H&E-stained images from the histological analysis; **(B)** Representative Masson-stained images from the histological analysis.

**FIGURE 9:**
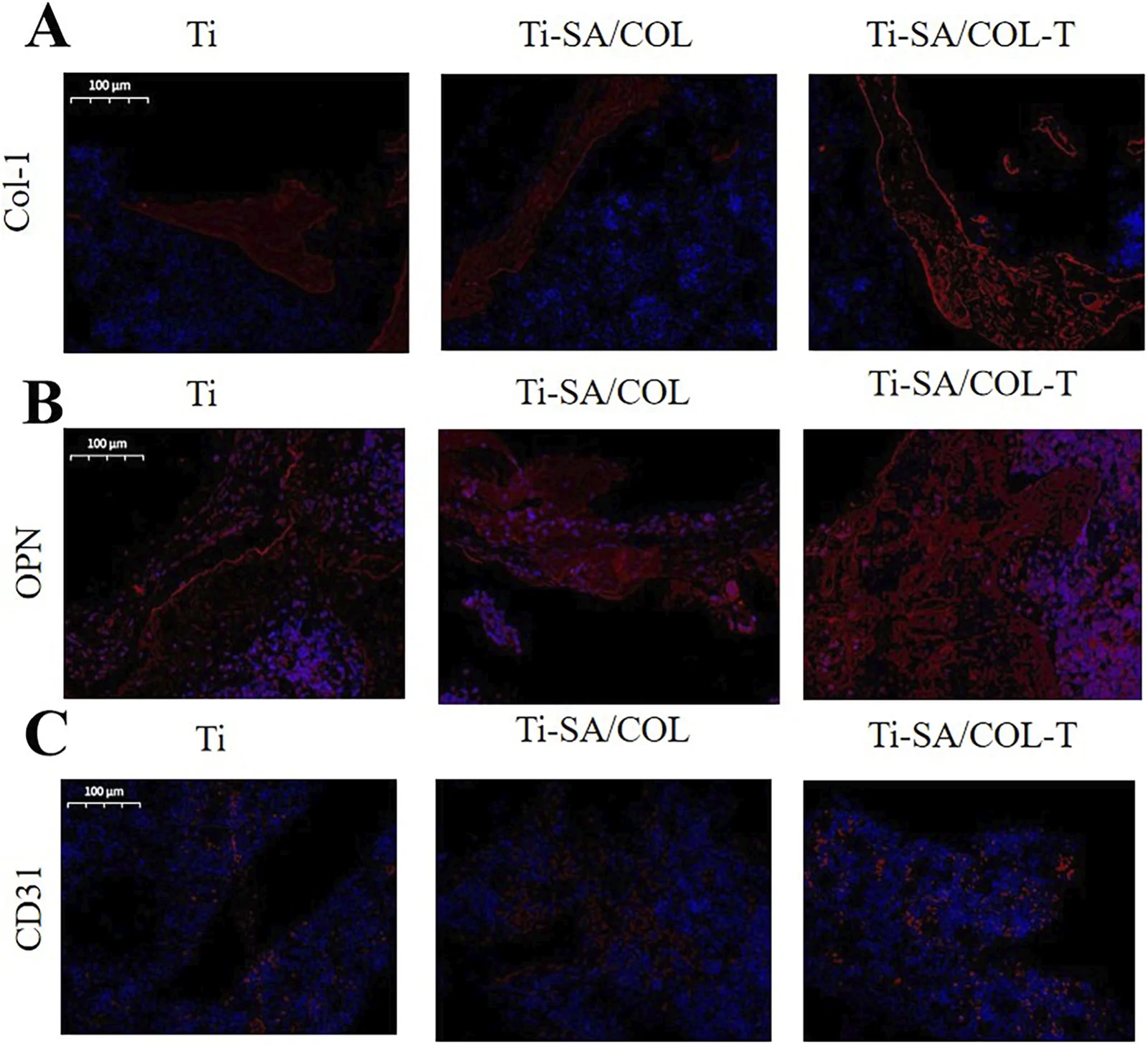
**(A)** Representative immunofluorescence images of Col-1; **(B)** Representative immunofluorescence images of OPN; **(C)** Representative immunofluorescence images of CD31.

## 4 Discussion

The results presented in our study illustrated that the alginate/collagen/troxerutin coating used in our study has an active effect on osteoblastic markers, which is beneficial to osseointegration. The ability to induce osteogenic differentiation was a key factor in improved titanium. We modified the titanium surface using layer-by-layer technology. Layer-by-layer method was a very reliable method for material preparation (Ghasemi et al., 2026; Sahebalzamani et al., 2026), alginate and collagen could be used for layer-by-layer method on the surface of titanium (Che et al., 2024). In our study, troxerutin was released from the coated surfaces on the titanium, where troxerutin can exert osteogenic and angiogenic effects. Polyphenolic compounds are a general term for a group of chemical substances in plants. There are more than 6,000 kinds of polyphenolic compounds in plants, including in fruits and vegetables. Polyphenolic compounds have antioxidation and anti-inflammatory effects and can reduce cardiovascular and cerebrovascular risks, improve gastrointestinal health, and enhance immunity (Jurikova et al., 2025). Polyphenolics are divided into four types: flavonoids, phenolic acids, tannins, and anthocyanins. Rabelo et al. (2025) reported that different kinds of flavonoids were beneficial to biomineralization. However, high doses of polyphenolics might have negative effects; thus, controlling the loading and release of polyphenolics is essential on the coated surface (Duda-Chodak and Tarko, 2023). Complex drug delivery systems may have problems with biocompatibility. In our study, this simple drug delivery system on the coated surface has satisfactory biocompatibility.

Troxerutin is a natural flavonoid. Owing to its excellent bioavailability and safety, troxerutin is widely used in clinical medicine. Few studies have confirmed the function of troxerutin in the skeletal system. Troxerutin promoted osteogenesis by reducing osteoclastogenesis. Yang et al. (2018) reported that troxerutin increased the expression of Osterix and Runt-related transcription factor 2, which could improve the mineralization of MC3T3-E1 cells. Wei et al. (2025) reported that troxerutin enhanced the osteogenic ability and biocompatibility of MC3T3-E1 cells and could also improve osteointegration and osteoconductivity *in vivo*. Yang et al. (2018) reported that troxerutin activated Wnt/β-catenin signaling, stimulating fracture healing and new bone formation. In our study, MC3T3-E1 cells were used to evaluate the mineralization of this material. We found that sodium alginate and collagen-loaded troxerutin improved the osteogenic ability of titanium.

Troxerutin produces vasculotropic and vasculoregulatory molecules and can enhance the angiogenic ability of vascular endothelial cells (Casili et al., 2021). Chronic venous insufficiency leads to severe socioeconomic and clinical impacts, and troxerutin is an effective treatment for chronic venous insufficiency (Miguel et al., 2025; Ma et al., 2019).

The surface morphology of the coating plays an essential role in enhancing the osteointegration of implants. We observed a porous structure in the SEM image of Ti-SA/COL, which provided an ideal environment for troxerutin loading. The water contact angle significantly decreased after surface modification, indicating that better hydrophilicity was observed in the Ti-SA/COL-T group, which was beneficial for osseointegration and primary stability.

Although this alginate/collagen/troxerutin coating promoted bone regeneration around the implants, it is still far from clinical application. First, the bioavailability of troxerutin is relatively low, and maintaining its stability within the body is difficult. Second, sterilizing this material after assembly is a troublesome issue. Many sterilization methods may destroy the coating, and a suitable sterilization process is required before the material is brought to market.

## 5 Conclusion

In conclusion, in this study, an alginate/collagen/troxerutin coating was evaluated, and we successfully loaded troxerutin on titanium and found that the osteogenic and angiogenic ability of the titanium was satisfactory. This coating is promising for future applications.

## Data Availability

The datasets presented in this study can be found in online repositories. The names of the repository/repositories and accession number(s) can be found in the article/Supplementary Material.
