# Supplementary material for: Sodium alginate and collagen-loaded troxerutin improve mineralization and vascularization around implants
Source: Front Bioeng Biotechnol. 2026 Oct 19;14:1935336. doi: 10.3389/fbioe.2026.1935336 (PMC13725097; doi:10.3389/fbioe.2026.1935336)
Supplement: Supplementary file 1 [file Table1.docx]

**Table S1**: Mouse PCR primer sequences of MC3T3-E1 cells used in this study

| **Gene** | **Forward primer (5 ′ -3 ′)** | **Reverse primer (5 ′ -3 ′)** |
| --- | --- | --- |
| GADPH | CTCGTCCCGTAGACAAAATGGT | GAGGTCAATGAAGGGGTCGTT |
| RUNX2 | AGAGTCAGATTACAGATCCCAGG | TGGCTCTTCTTACTGAGAGAGG |
| ALPL | GAACAGAACTGATGTGGAATACGAA | CAGTGCGGTTCCAGACATAGTG |
| COL1A1 | GATGTTGAACTTGTTGTTGCTGAGGG | GGCAGGCGAGATGGCTTATT |
| BGLAP | GAACAGACAAGTCCCACACAGC | TCAGCAGAGTGAGCAGAAAGAT |

**Table S2**: Human PCR primer sequences of HUVECs cells used in this study

| **Gene** | **Forward primer (5 ′ -3 ′)** | **Reverse primer (5 ′ -3 ′)** |
| --- | --- | --- |
| GADPH | TCAAGAAGGTGGTGAAGCAGG | AGCGTCAAAGGTGGAGGAGTG |
| VEGF | AGGGCAGAATCATCACGAAGT | AGGGTCTCGATTGGATGGCA |
